# Histamine Regulates Molecular Clock Oscillations in Human Retinal Pigment Epithelial Cells *via* H_1_ Receptors

**DOI:** 10.3389/fendo.2018.00108

**Published:** 2018-03-19

**Authors:** Eri Morioka, Yuzuki Kanda, Hayato Koizumi, Tsubasa Miyamoto, Masayuki Ikeda

**Affiliations:** ^1^Graduate School of Science and Engineering, University of Toyama, Toyama, Japan

**Keywords:** antihistamine, cytosolic calcium, human, molecular clock, retina, transcriptional regulation

## Abstract

Vertebrate eyes are known to contain circadian clocks, but their regulatory mechanisms remain largely unknown. To address this, we used a cell line from human retinal pigment epithelium (hRPE-YC) with stable coexpression of reporters for molecular clock oscillations (*Bmal1-luciferase*) and intracellular Ca^2+^ concentrations (*YC3.6*). We observed concentration-dependent increases in cytosolic Ca^2+^ concentrations after treatment with histamine (1–100 µM) and complete suppression of histamine-induced Ca^2+^ mobilizations by H_1_ histamine receptor (H_1_R) antagonist *d*-chlorpheniramine (*d*-CPA) in hRPE-YC cells. Consistently, real-time RT-PCR assays revealed that H_1_R showed the highest expression among the four subtypes (H_1_–H_4_) of histamine receptors in hRPE-YC cells. Stimulation of hRPE-YC cells with histamine transiently increased nuclear localization of phosphorylated Ca^2+^/cAMP-response element-binding protein that regulates clock gene transcriptions. Administration of histamine also shifted the *Bmal1-luciferase* rhythms with a type-1 phase-response curve, similar to previous results with carbachol stimulations. Treatment of hRPE-YC cells with *d*-CPA or with more specific H_1_R antagonist, ketotifen, blocked the histamine-induced phase shifts. Furthermore, an H_2_ histamine receptor agonist, amthamine, had little effect on the *Bmal1-luciferase* rhythms. Although the function of the *in vivo* histaminergic system within the eye remains obscure, the present results suggest histaminergic control of the molecular clock *via* H_1_R in retinal pigment epithelial cells. Also, since *d*-CPA and ketotifen have been widely used (e.g., to treat allergy and inflammation) in our daily life and thus raise a possible cause for circadian rhythm disorders by improper use of antihistamines.

## Introduction

The histaminergic system in the central nervous system controls diverse physiological functions including sleeping–waking, thermoregulation, and feeding (1). To achieve these functions, histaminergic neurons in the tuberomammillary nucleus (TMN) of the posterior hypothalamus send long-distance axons into diverse brain areas (2). Interestingly, histaminergic projections from the brain to the retina have been shown to exist in rodents and primates (3–6), but knowledge on their physiological functions remains limited. In baboon eyes, histamine reduced flash sensitivity in ON ganglion cells (7). In macaque eyes, H_1_ histamine receptor (H_1_R) was expressed in horizontal cells, and H_2_ histamine receptor (H_2_R) was expressed in cone photoreceptors (8). Histamine significantly reduced hyperpolarization-activated currents recorded from cones in monkeys (8) and modulated retinal ganglion cell firings in rats and monkeys (9). Furthermore, dopaminergic amacrine cells in mice expressed H_1_R and displayed histamine-induced cytosolic calcium mobilizations (10). Source of histamine within the retina may not be only from the TMN projections but also from local synthesis because genes encoding histamine synthetic enzyme, histidine decarboxylase (HDC), were expressed in the outer nuclear layer of mice retina (11). Meanwhile, no apparent changes in retinal structures and functions were identified in HDC knockout (HDC^−/−^) mice (11), and thus retinal histaminergic regulations remain controversial.

**Figure 1:**
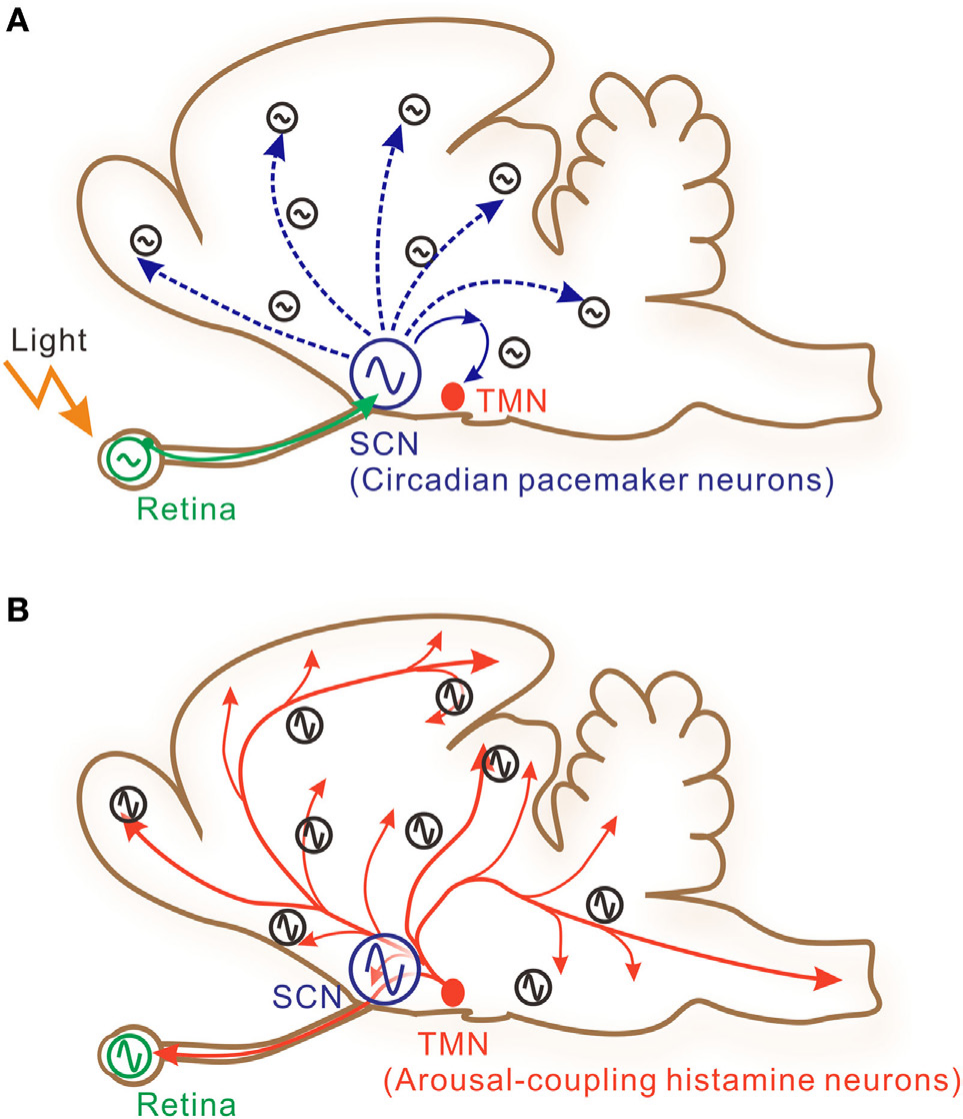
A schematic illustration of interactive signaling underlying photic and histaminergic regulation of the circadian clock. **(A)** The circadian pacemaker neurons located within the hypothalamic suprachiasmatic nucleus (SCN) are directly controlled by retinal projections (solid green arrow). The retina contains independent circadian oscillators controlling photic sensitivity. Clock gene oscillations are observed in many other cells in the brain, although system-level oscillations are strongly dependent on core SCN oscillations. These peripheral clock oscillations are controlled by indirect neural connections (dashed blue arrows) or humoral factors from the SCN. The tuberomammillary nucleus (TMN) of the posterior hypothalamus, which contains histamine neurons, may also be under the control of the SCN *via* hypothalamic neural networks. **(B)** The TMN sends long-distance axons directly to diverse brain areas and to the retina (solid red arrows). SCN neurons and retinal cells also receive histaminergic fibers and may be under the control of the TMN. Taken together, these features suggest that histaminergic signaling may function as a feedback signal to stabilize or boost system-wide circadian oscillations.

Histamine release from histaminergic neurons is generally coupled with vigilance states, being active during wakefulness and inactive during sleep (1, 12). Daily rhythms of sleep and wakefulness are strongly regulated by the central circadian pacemaker located within the hypothalamic suprachiasmatic nucleus (SCN; Figure 1A) (13). Meanwhile, SCN neuronal activity rhythms are directly regulated by histaminergic projections (1, 14). This suggests the presence of a histaminergic feedback system between the SCN clock and histaminergic sleep–wake mechanisms (Figure 1B). In addition, it is well known that the mammalian retina contains a circadian clock, because rhythmic clock gene expressions have been reported in various retinal cells (15–18). It has also been shown that photoreceptor disk shedding (19–21), dopamine synthesis (22), melatonin release (23), and retinal electrical responses to light (24) are all under circadian clock control. SCN neurons were reported to receive axons from intrinsically photosensitive retinal ganglion cells for photoentrainment of circadian rhythms (25). Thus, it is reasonable to hypothesize that the histaminergic system within the eye may function as an additional feedback system that intermediates between the retinal and central circadian clocks (Figure 1B). However, substantial evidence is lacking to prove this hypothesis. In the earlier works, circadian rhythms in clock gene transcriptional levels and adenylyl cyclase activities were identified in retinal pigment epithelial (RPE) cells (26–28). Based on these findings, a cell line was recently generated from human retinal pigment epithelium, hRPE-YC (29), that stably coexpresses reporters for clock gene transcriptions (*Bmal1-luciferase*) and intracellular Ca^2+^ concentrations (*YC3.6*). Using this model cell line, this study provides evidence for functional expression of H_1_R and histaminergic control of the molecular clock within the eye.

## Materials and Methods

### Cell Cultures

hRPE-YC cells (less than five passages) were cultured in Dulbecco’s modified Eagle’s medium/F12 supplemented with 10% fetal bovine serum (Invitrogen, Carlsbad, CA, USA), sodium bicarbonate (1.2 g/L), and 1% penicillin/streptomycin antibiotics (Invitrogen) under constant temperature (37°C) and 5% CO_2_.

### Ca^2+^ Imaging

The Ca^2+^ imaging protocols were described previously (30). Briefly, cells were seeded onto 35-mm glass-bottom dishes. The culture medium was gently rinsed from the dishes using buffered salt solution. Fluorescence images were obtained under perfusion of buffered salt solution using an upright microscope (Axioplan 2; Carl Zeiss, Thornwood, NY, USA) with a water-immersion objective (Achroplan ×20 NA0.5w; Carl Zeiss). Pairs of fluorescent images (535 ± 15/480 ± 15 nm) were produced with a light pulse of 440 ± 5 nm generated by a dual filter wheel system (Lambda 10-3; Sutter Instruments, Novato, CA, USA) and acquired using a cooled charge-coupled device camera (CoolSnap Fx; Photometrics, Tucson, AZ, USA). The timings of shutter gating and image acquisitions at 6-s intervals were regulated by digital imaging software (MetaFluor ver. 6.0; Japan Molecular Devices, Tokyo, Japan). Histamine and *d*-chlorpheniramine (*d*-CPA) (both from Sigma-Aldrich, St. Louis, MO, USA) were perfused onto the cells by switching the perfusate.

### *Bmal1-Luciferase* Assay

The *Bmal1-luciferase* rhythms were analyzed as described (29) using culture medium supplemented with 50 µM beetle luciferin (Promega, Madison, WI, USA) and a multichannel chemiluminescence analyzer (Kronos-Dio, Model AB-2550; ATTO Co. Ltd., Tokyo, Japan) set at 37°C. The time point with the peak chemiluminescence level in the *Bmal1-luciferase* rhythms was regarded as circadian time (CT) 20. To analyze phase-response curves (PRCs) against pharmacological stimulations, Kronos recordings were paused for 5 min. During the pause, 10% of culture medium (100 µL) was collected from each dish. Histamine, amthamine dihydrobromide, ketotifen fumarate (Sigma-Aldrich), or *d*-CPA was added to the collected culture medium and gently returned to the culture dish (final diluted concentration: 50 µM for histamine, 50 µM for amthamine dihydrobromide, 10 µM for *d*-CPA, and 10 µM for ketotifen). Although hRPE-YC cells represented little sensitivity to light (29), above medium exchanges were carefully conducted under dim red light (<3 lx). The PRCs were eye fitted by three experienced investigators.

### Immunofluorescence Confocal Imaging

To examine the effects of histamine on the phosphorylation levels of Ca^2+^/cAMP response element-binding protein (CREB), hRPE-YC cells plated on 35-mm glass-bottom dishes were stimulated with histamine (100 µM) for 10 min during subjective night-time. Immediately after the stimulations, hRPE-YC cells were fixed in 4% phosphate-buffered paraformaldehyde for 15 min and washed three times with phosphate-buffered saline. The samples were immunostained with 1:100 diluted affinity-purified rabbit anti-P-CREB (pSer^133^) (Sigma-Aldrich) and embedded in Vectashield (Vector Laboratories, Burlingame, CA, USA) containing 4′,6-diamidino-2-phenylindole (DAPI) as described (29). Images were acquired using a confocal laser-scanning microscope (A1MP plus; Nikon, Tokyo, Japan).

### Real-Time RT-PCR Assay

The mRNAs for the four histamine receptor subtypes (H_1_–H_4_) and HDC were quantified by referring a housekeeping gene (human β*-actin*) in hRPE-YC cells using a real-time RT-PCR system (Roter-Gene Ver. 6.0 software, Corbett Research, Sydney, NSW, Australia). The cell cultures and RNA extraction procedures were described previously (29). The PCR primers for histamine receptors (31) and HDC (32) were designed elsewhere. Each primer (100 µM) was used with Rotor-Gene SYBR Green RT-PCR Master Mix (Qiagen, Germantown, MD, USA) in the 72-well rotor of the PCR system (Rotor Gene 3000A; Corbett Research) as described (29). mRNA levels were expressed as 2^−ΔCt^ using β*-actin* mRNA level as internal standard.

### Statistical Analysis

Data are presented as mean ± SEM. One-way analysis of variance followed by Duncan’s multiple range test and four-parameter Hill function were used to analyze concentration–response curve for histamine. Kruskal–Wallis test followed by Steel–Dwass test was used to compare gene expression profiles. A two-tailed Student’s *t*-test was used for pairwise comparisons. A 95% confidence level was considered to indicate statistical significance.

## Results

**Figure 2:**
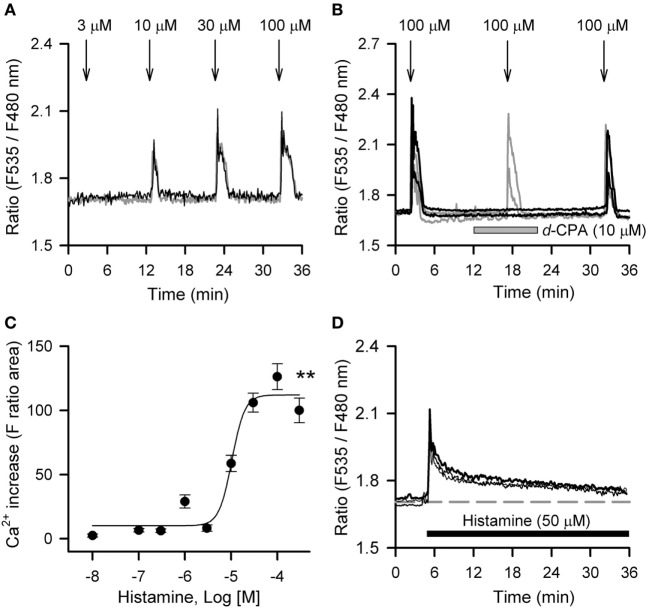
Histamine-induced calcium mobilizations in hRPE-YC cells. **(A)** Histamine (3–100 µM) evoked dose-dependent increases in cytosolic Ca^2+^ concentrations in hRPE-YC cells. Two representative cell responses are shown. Arrows denote the onsets of 1-min histamine stimulations. **(B)** Repeated 100-µM histamine stimulations with 15-min intervals consistently elevated cytosolic Ca^2+^ in hRPE-YC cells (gray traces). In this experiment, the second histamine stimulation was also examined under perfusion of 10 µM *d*-chlorpheniramine (*d*-CPA) (black traces). Note that complete inhibition of Ca^2+^ responses by *d*-CPA and recovery of Ca^2+^ responses after rinsing out of *d*-CPA were observed. All of the above experiments were reproducible in at least three independent trials in separate culture dishes. **(C)** Concentration-response curves for histamine. **P < 0.01 by one-way analysis of variance. **(D)** Continuous perfusion of histamine (50 µM, black bar) mobilized Ca^2+^ depending on the onset of stimulations without further amplification of Ca^2+^ responses during stimulations. The gray dashed line denotes the mean baseline Ca^2+^ level in the three representative cells.

Histamine mobilized intracellular Ca^2+^ in hRPE-YC cells in a concentration-dependent manner with an EC_50_ value of 10.4 µM (Figures 2A–C). At 100 µM, histamine consistently evoked a Ca^2+^ response in nearly all cells tested (97 ± 1.4%; 397 of 410 cells in 18 dishes). The histamine-induced Ca^2+^ response was significantly inhibited by pretreatment with 10 µM *d*-CPA (Figure 2B), suggesting that the response was primarily mediated by H_1_R. Intracellular Ca^2+^ mobilizations after continuous bath application of 50 µM histamine were also examined, because this application was used for the *Bmal1-luciferase* assays. Bath application of 50 µM histamine produced a transient Ca^2+^ response that almost recovered to the baseline during a 30-min exposure period (Figure 2D).

**Figure 3:**
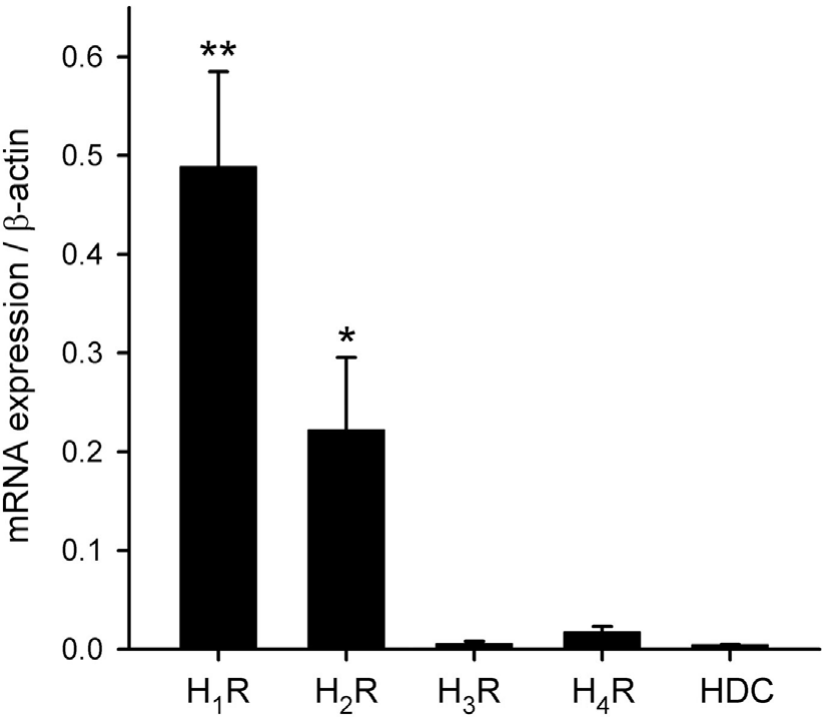
Transcriptional profiles of histamine receptor subtypes and histamine synthetic enzyme. H_1_–H_4_Rs and histidine decarboxylase (HDC) mRNAs were quantified in hRPE-YC cells by quantitative RT-PCR with 2^−ΔCt^ using β-actin as internal control. H_1_ histamine receptor (H_1_R) exhibited the highest expression among the four subtypes. There were also detectable levels of H_2_ histamine receptor (H_2_R) expression in these cells. HDC was not expressed in hRPE-YC cells. **P < 0.01 and *P < 0.05 by Kruskal–Wallis test followed by Steel–Dwass test. Data represent mean ± SEM from 11 dishes.

To characterize the histamine receptor subtypes (H_1_–H_4_) expressed in hRPE-YC cells, cells were collected for real-time RT-PCR assays at early subjective daytime (CT1–CT3; number of dishes = 7) and early subjective night-time (CT13–CT15; number of dishes = 4) following on-line monitoring of the *Bmal1-luciferase* rhythms. The results revealed that H_1_R showed the highest expression among the four histamine receptor subtypes with no difference in the levels between subjective daytime and subjective night-time. The expression levels of H_3_ and H_4_ histamine receptors were near to the detection limits. Therefore, the gene expression levels were further analyzed regardless of sampling time using a non-parametric test (Figure 3). The expression levels of H_1_R (*P* < 0.01) and H_2_R (*P* < 0.05) were significantly larger than those of H_3_ and H_4_ histamine receptors (Kruskal–Wallis test followed by Steel–Dwass test). In addition to these analyses, capability of histamine synthesis in hRPE-YC cells was analyzed by monitoring HDC expression. However, HDC expression was negligible in these cells (Figure 3).

**Figure 4:**
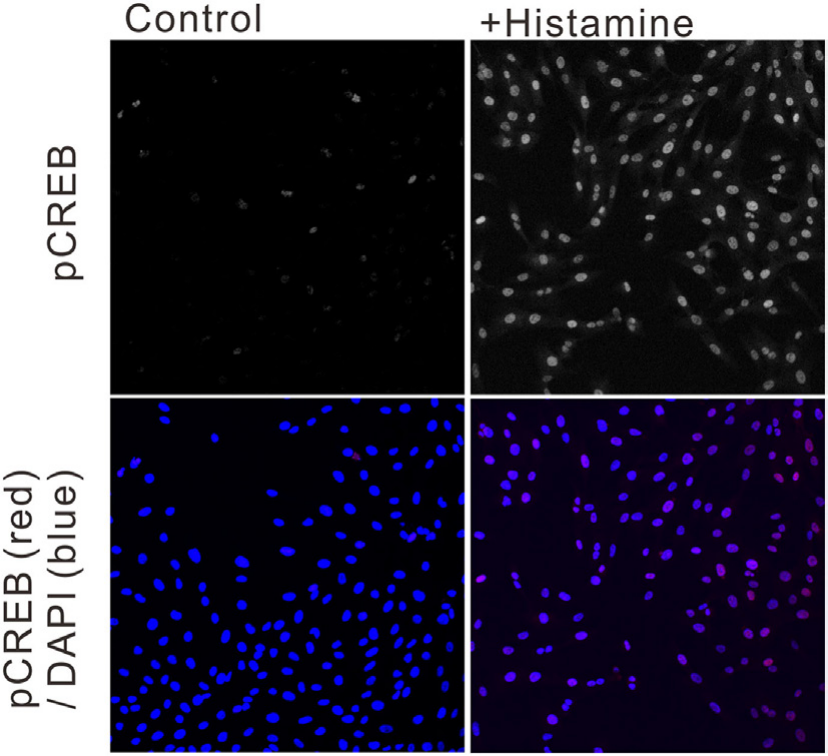
Ca^2+^/cAMP-response element-binding protein phosphorylation following histamine exposure. Immunofluorescence staining of phosphorylated CREB (pCREB) in unstimulated control hRPE-YC cells (left two images) and hRPE-YC cells after exposure to 50 µM histamine for 10 min (right two images). Counter-staining with 4′,6-diamidino-2-phenylindole (blue color in merged picture) demonstrated the nuclear localization of pCREB signals in hRPE-YC cells following the histamine stimulation.

**Figure 5:**
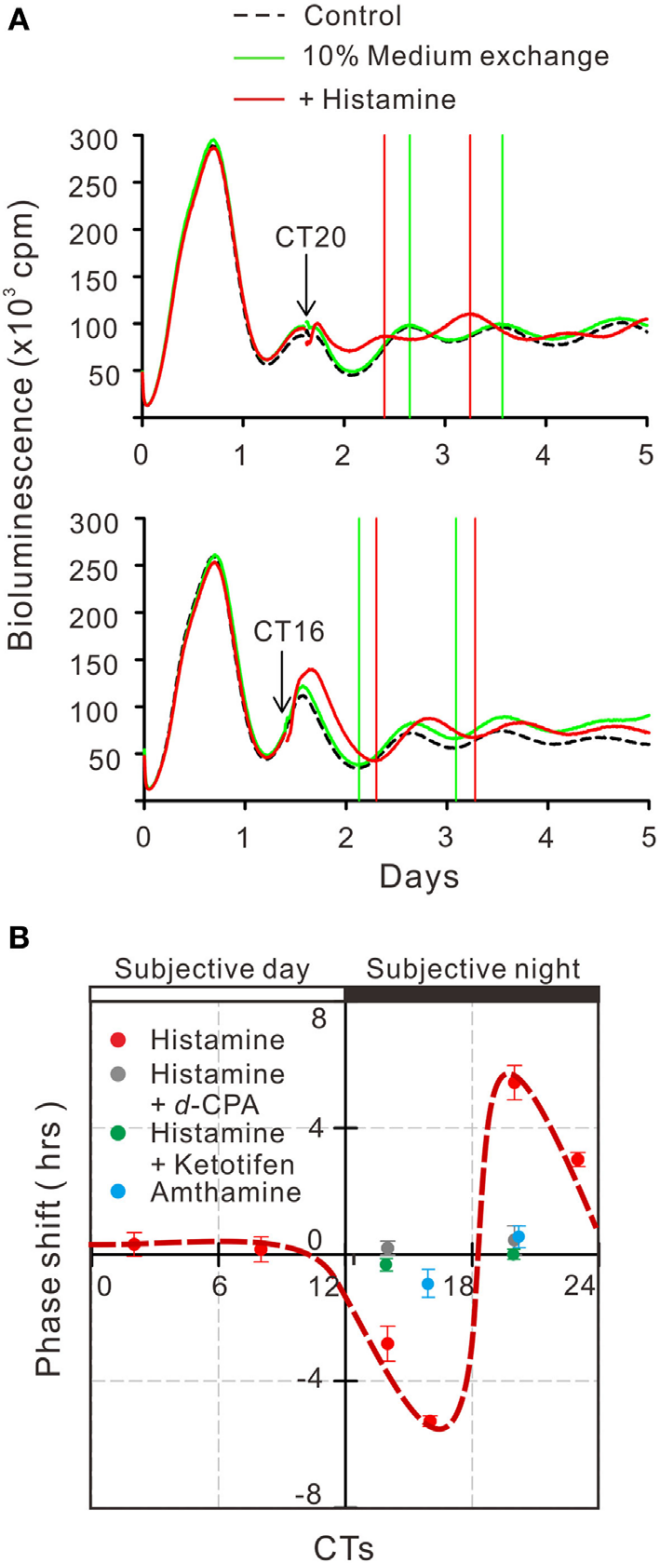
Circadian phase shifts in *Bmal1* transcriptional rhythms following histamine exposure. **(A)** The mean *Bmal1-luciferase* intensities in 35-mm dishes were quantified using a multichannel chemiluminescence analyzer. The arrows indicate onset of 50 µM histamine exposures at CT20 and CT16. Subsequent troughs or peaks of circadian waves were compared with groups of non-treated control cells. **(B)** Based on the histamine-induced phase shifts at various time points, a type-1 phase-response curve was eye fitted (red circles with dotted line). Similar stimulation of cells by the H_2_ histamine receptor agonist amthamine at CT20 or CT16 produced significantly smaller phase shifts (blue circles). Treatment with 10 µM *d*-chlorpheniramine (*d*-CPA) or ketotifen almost completely abolished the histamine-induced phase-advances at CT20 and delays at CT14 (gray circles for *d*-CPA treatment and green circles for ketotifen treatment). Data represent mean ± SEM from three to six dishes.

To estimate the gene transcriptional regulations by histamine, immunofluorescence staining of phosphorylated CREB (pCREB) in hRPE-YC cells after histamine stimulation was examined. Compared with unstimulated controls (optical density = 31 ± 0.2, number of cells = 328, number of dishes = 3), treatment with 50 µM histamine for 10 min doubled the pCREB staining levels in the nucleus (optical density = 65.5 ± 1.9, number of cells = 312, number of dishes = 3; Figure 4). Consistent with the nuclear pCREB inductions, the same histamine stimulation produced circadian phase-delays or advances in the *Bmal1-luciferase* rhythms (Figure 5A). Accordingly, the type-1 PRC was eye fitted on the phase-shifting profiles dependent on the CT (Figure 5B). The histamine-induced phase shifts at CT14 (−2.8 ± 0.6 h, number of dishes = 5 in histamine-stimulated group) and CT20 (+5.4 ± 0.5 h, number of dishes = 6 in histamine-stimulated group) were almost completely inhibited by *d*-CPA or ketotifen treatment (Figure 5B). Compared with the phase responses to histamine stimulations, the H_2_R-specific agonist amthamine (50 µM) produced smaller phase delays (18.6% of histamine responses, *P* < 0.01 by Student’s *t*-test, number of dishes = 3 at CT16) and phase advances (10.3% of histamine responses, *P* < 0.01 by Student’s *t*-test, number of dishes = 3 at CT20; Figure 5B).

## Discussion

In this study, we explored the functions of histamine signaling in RPE cells using a human cell line. The results for Ca^2+^ imaging and real-time RT-PCR clearly demonstrated functional expression of H_1_R in hRPE-YC cells. As H_1_R couples with G_q_ proteins and links with the phosphatidylinositol signaling pathway to mobilize cytosolic Ca^2+^, the conventional intracellular signaling pathway reported for RPE cells (33, 34) could be the trigger for phase shifts of *Bmal1* transcriptional rhythms. Indeed, nuclear pCREB expression was observed following the histamine stimulations. These results are consistent with a previous finding that stimulation of G_q_-coupled *M3* muscarinic acetylcholine receptors in hRPE-YC cells resulted in transient Ca^2+^ increases, nuclear pCREB expressions, and phase shifts of *Bmal1-luciferase* rhythms with a type-1 PRC (29). Meanwhile, this study indicated gene expression of G_s_-coupled H_2_R in hRPE-YC cells but failed to demonstrate apparent phase shifts of *Bmal1-luciferase* rhythms by amthamine. In the previous study, forskolin, a pharmacological activator of adenylate cyclase, produced apparent phase shifts in hRPE-YC cells (29). Taken together, it is suggested that functional H_2_R expression and activation of the downstream adenylate cyclase pathway could be limited in hRPE-YC cells. Histamine-induced circadian phase shifts have been studied in SCN slice preparations by reference to action potential firing rhythms (14), and this study indicates that similar histaminergic regulations may be present in the retinal circadian clock.

Retinal pigment epithelial cells have multiple functions within the retina. Among these, it should be emphasized that RPE cells are involved in the daily photoreceptor disk shedding critical for circadian rhythms in photic sensitivities. Phagocytosis of the photoreceptor outer segment by RPE cells is directly triggered by light or by intrinsic circadian clock mechanisms given that the rhythm is sustained under constant darkness (21). Although innervations from central histaminergic neurons have been identified primarily in the ganglion cell layer and inner plexiform layer in the retina (4–9), histamine could be a paracrine modulator for various retinal cells. In addition, it has shown that the outer nuclear layer of mice retina express HDC genes using a laser microdissection technique (11), although the type of retinal cells synthesizing histamine has not yet been characterized. Thus, it is possible that histaminergic control of RPE cells, if any in *in vivo*, could be involved in the regulation of photoreceptor disk shedding rhythms. It was also shown that HDC^−/−^ mice with recovery of the *Crb1* mutation exhibit normal retinal structures and functions, including the outer segment (11). However, these analyses were conducted under 12-h/12-h light/dark cycles and paid no particular attention to the tissue sampling time. Under these circumstances, the direct light information was presumably sufficient to determine the phenotypes. Histamine release from histaminergic neurons is coupled with sleep–wake states (1, 12). Importantly, significant reductions in clock gene (*Per1* and *Per2*) transcriptional rhythms have been shown in many brain regions outside the SCN in HDC^−/−^ mice (35). This suggests remote control of peripheral clock gene transcriptional rhythms by the brain histaminergic system (Figure 1). Thus, it is of particular interest whether changes in histaminergic tones and sleep–wake status can exert feedback on retinal clock regulations and ultimately on circadian clock systems. Further studies are needed to clarify these possibilities.

In relation to the effect of the H_1_R antagonist observed in this study, we would like to emphasize the possible influence on human circadian clock regulations because H_1_R antagonists are widely used in daily life. First-generation H_1_R antagonists, such as *d*-CPA, are permeable to the brain and induce sedation and/or slow-wave sleep following systemic administration in rats (36, 37). Based on these effects, one of the first-generation H_1_R antagonists, diphenhydramine, is currently sold as a sleeping aid in Japan. In addition, doxepin, another first-generation H_1_R antagonist known to induce sleep, has been approved by the FDA for treatment of insomnia in the United States (38). Furthermore, numerous H_1_R antagonists are currently sold as eye drops to treat ocular allergies (39). Despite the widespread use of H_1_R antagonists, their influence on circadian clock regulations has not been analyzed in detail. The effects of daily systemic injections of ketotifen (an early phase second-generation H_1_R antagonist) were recently evaluated in rats, with significant effects observed on their circadian locomotor activity rhythms (40). In addition, we preliminary observed reduction in *Per2* transcriptional rhythms in the SCN and hippocampus by daily systemic injections of ketotifen in rats (unpublished data). Numerous antihistamines, including *d*-CPA and ketotifen, represent affinity to muscarinic receptors to block acetylcholine signaling (41, 42) and thus use of antihistamines especially at high doses may also exert their influence on cholinergic clock regulations (29, 43). Together with the present results showing complete suppression of histamine-induced circadian phase shifts in hRPE-YC cells by *d*-CPA or ketotifen, we suggest that further clinical studies to analyze the influence of antihistamines on human circadian rhythms, with a special focus on circadian visual functions, are warranted.

In conclusion, the present results suggest histaminergic control of the molecular clock *via* H_1_R in a model cell line for human RPE cells and thus raise a possible cause for circadian rhythm disorders by daily use of antihistamines.

## Author Contributions

MI designed the study, wrote and edited the manuscript, and directed the project. EM, YK, HK, and TM performed the experiments. EM analyzed the data.

## Conflict of Interest Statement

The authors declare that the research was conducted in the absence of any commercial or financial relationships that could be construed as a potential conflict of interest.

## Abbreviations

ANOVA, analysis of variance; CPA, chlorpheniramine; CREB, Ca^2+^/cAMP-response element-binding protein; CT, circadian time; H_1_R, H_1_ histamine receptor; H_2_R, H_2_ histamine receptor; HDC, histidine decarboxylase; DAPI, 4′,6-diamidino-2-phenylindole; pCREB, phosphorylated CREB; PRC, phase-response curve; RPE, retinal pigment epithelial; SCN, suprachiasmatic nucleus; TMN, tuberomammillary nucleus.
